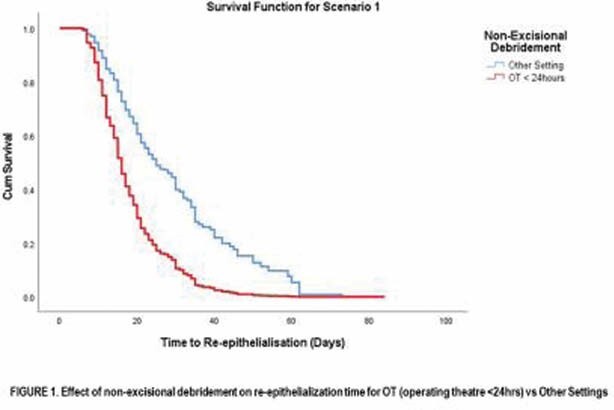# 84 Effects of Early, Non-excisional Debridement on Pediatric Burn Wound Re-epithelialisation Time

**DOI:** 10.1093/jbcr/irac012.087

**Published:** 2022-03-23

**Authors:** Bronwyn R Griffin, Anjana Bairagi, Maleea Holbert, Roy M Kimble

**Affiliations:** Griffith University, South Brisbane, Queensland; Queensland Children's Hospital, Brisbane, Queensland; Children's Hospital Queensland, South Brisbane, Queensland; Children's Hospital Queensland, South Brisbane, Queensland

## Abstract

**Introduction:**

Reported advantages of early excision for larger burn injuries include reduced morbidity, mortality, and hospital length of stay for adult burn patients. However, a paucity of evidence supports the best option for paediatric burns and the advantages of non-excisional (mechanical) debridement. This study aims to evaluate the association between early (< 24hours post-injury) non-excisional debridement under general anaesthesia with burn wound re-epithelialisation time and skin graft requirements.

**Methods:**

A cohort study of children (< 17 years) presenting with burns >5% total body surface area, using prospectively collected state-wide pediatric burns' registry between January 2013 to December 2019. Primary outcomes were: time to reepithelialization (tested using survival analysis) and skin graft requirements, tested using binary logistic regression for odds ratios). Using depth and size, we performed a propensity matched dataset to analyse effects of early non-excisional debridement in the operating theatre.

**Results:**

Overall, 392 children met eligibility (males 58.2%). When propensity matched, early non-excisional debridement under general anaesthesia in the operating theatre, significantly reduced the time to re-epithelialisation (15.0 (CI: 11.00-20.00) versus 20.0 (CI:13.5 – 31.00) days) and the odds of requiring a skin graft (OR:0.319 (0.125 – 0.812).

**Conclusions:**

This study is the first to demonstrate that early non-excisional debridement under general anaesthesia in the operating theatre significantly reduces wound re-epithelialisation time and subsequent need for a skin graft in paediatric burn patients. Analysis suggests that ketamine procedural sedation and analgesia in the emergency department used for burn wound debridement is not an effective substitute for debridement in the operating theatre.